# New challenges in dietary pattern analysis: combined dietary patterns and calorie adjusted factor analysis in type 2 diabetic patients

**DOI:** 10.1186/2251-6581-13-71

**Published:** 2014-07-10

**Authors:** Zhaleh Shadman, Mahdieh Akhoundan, Nooshin Poorsoltan, Bagher Larijani, Mostafa Qorbani, Mohsen Khoshniat Nikoo

**Affiliations:** 1 Endocrinology and Metabolism Research Center, Endocrinology and Metabolism Clinical Science Institute, Tehran University of Medical Sciences, Tehran, Iran

**Keywords:** Factor analysis, Diabetes mellitus, Type 2

## Abstract

**Background:**

Some variability for dietary pattern analysis due to subjective procedures (e.g. arbitrary food categorization and number of factors extraction) was reported. The aim of this study was to present or design a new approach to challenge the conventional dietary pattern analysis through new classification of dietary patterns according to the possibility of the high adherence to more than one dietary pattern and calorie adjusted factor extracting.

**Methods:**

This cross-sectional study conducted on 734 type2 diabetic patients. Factor analysis defined three major dietary patterns (Western like, Asian like and Traditional like) and the associations of each pattern were assessed with glycemic control and lipid profiles among tertiles of each pattern. In order to compare variables in highest tertile of three defined dietary patterns, eight new different groups were classified according to the high adherence to one or more patterns and ANOVA and ANCOVA were used to compare them. Also, calorie adjusted factor extracting were done to find out if the same factor loadings would be extract.

**Results:**

Among three major dietary patterns, only Western like showed a significant association with fasting blood sugar (p = 0.03, 12.49 ± 5.99), serum total cholesterol (p = 0.02, 8.71 ± 3.81) and LDL cholesterol (p = 0.04, 5.04 ± 2.40). While comparison of new classified patterns, showed no significant differences, except a high blood glucose in Western like- Asian like versus traditional like dietary pattern (p = 0.04). Also, calorie adjusted factor extracting showed different factor loadings.

**Conclusions:**

Results showed that the conventional dietary pattern analysis method may have substantial limitations in interpreting the results and may lead to inappropriate conclusions.

## Background

Factor analysis method in nutritional studies derived from the thought that considering a group of nutrients together in comparison to studying each food or nutrient separately, may parallel more closely the real world [1]. In this method, each participant would be given a score according to adherence level of all derived dietary patterns for example Western, Prudent and Mediterranean. Although such a method could be affected by several subjective or arbitrary decisions in variable reduction or decision to consideration of number of factors [2] as dietary patterns. But other inconsistencies or limitations may be hidden within such an analysis especially in the field of nutrition and dietary patterns. In conventional method, statistical analyses were done within each dietary pattern according to the level of adherence [1]. So, one question arises would be that is it possible a participant would have higher score in more than one defined dietary pattern? And if so, does assessment the adherence level of a single dietary pattern regardless of considering the level of adherence to other dietary patterns would lead to correct answers? In other words, it was not done comparison of one dietary pattern with others, rather it was just done within one dietary pattern comparison, by dividing participants to several groups (according to the median, tertile, quartile, quantile and etc.). Also, another question is that when the studying population includes a vast range of dietary requirements, is it true extracting the factor loading on crude food intake? The aim of this study was to compare the results of conventional statistical analysis the new classification according to standing on highest adherence to one or more dietary patterns and also primary calorie adjusted factor loading.

## Methods

The Endocrinology and Metabolism Research Center ethic committee (E00192) approved this cross-sectional study. This cross-sectional study was conducted on 734 type 2 diabetic patients who have been followed by Diabetes and Metabolic disease Clinic of Tehran University of Medical Sciences. Inclusion criteria were age 35–65, diagnosis of diabetes after age of 30 and suffering from diabetes mellitus for more than 5 years, no changes in the use of drugs during the last year, the absence of insulin therapy, myocardial infarction, angina pectina, stroke, and acute liver or renal disease during the past year, chronic inflammation, thyroid disease, genital diseases, vegetarianism, alcohol consumption, smoking, and pregnancy. Each participant filled a consent form as their confirming information about the study procedere.

Patients were asked to fill a validated 168-item food frequency questionnaire (FFQ) [3] designed to assess food items consumed over the past year by face-to-face interviews. Data were analyzed using adjusted N4 software (Nutritionist: version 4.0; Tinuviel Software, Warrington, United Kingdom). Before analysis of dietary pattern, consumption frequencies of different food categories were transformed to serving per week frequencies. To identify dietary patterns, the 168 food items were categorized into 22 food groups based on their similarity of nutrient content and previous studies [4]. Height was measured with a wall-mounted stadiometer to the nearest 0.1 cm. Weight was determined to the nearest 0.1 kg on the same properly calibrated electronic digital scale, without shoes, with minimal clothing, and after voiding. Physical activity level (PAL) was assessed by a validated questionnaire defined by nine different metabolic equivalents. Venous blood was collected after overnight fasting for at least 12 h for biochemical analysis.

Serum glucose concentration was measured by fluorometric method according glucose oxidase principle (Glucose determination kit, Parsazmun, Tehran, Iran) through auto-analyzer instrument (Hitachi 902, Roche, Basel, Switzerland). Glycated hemoglobin was determined on whole blood sample by HbA1c Pink Kit and DS5 analyzer. Serum triglyceride, total cholesterol, LDL and HDL cholesterol were measured by the related biochemical kits (Parsazmun, Tehran, Iran) by the auto-analyzer (Hitachi 902, Roche, Basel, Switzerland).

### Statistical analysis

All statistical analyses were performed by SPSS software (version 16.0; SPSS Inc, Chicago). Dietary patterns were derived using principal component analysis (PCA) based on the 168 food items. Sampling adequacy and inter-correlation of variables were supported by KMO (Kaiser-Meyer-Olkin) value = 0.69 and Bartlett’s test of sphericity < 0.0001, respectively. Eigen values > 1.5 determined whether a factor should be considered as major dietary patterns. Varimax rotation was applied to review the correlations between variables and factors. Food groups with positive loadings in each pattern indicate the direct relationship and food groups with negative loadings shows the inverse relationship with that pattern. The factor score for each pattern was calculated by summing the consumption of each food group that were weighted by factor loading and each person received an individual factor score for each identified pattern [1]. Factor scores were then categorized into three groups based on the tertiles of factor scores. Linear regression models were used to assess the association of adherence to three major dietary patterns with the mean concentrations of serum glucose, lipid profiles and HbA1c. In addition, multivariate linear regression models including age, sex, diabetes duration, type and drug dosage and calorie intake were used to distinguish the possible effect of adherence to dietary patterns and other related factors. An alpha level of less than 0.05 was accepted in all tests as statistically significant and with the sample size of 734; a power value of 90% was generated.

To compare the dependant variables in highest tertile of three dietary patterns, a new classification was created in which, those who were not scored in highest tertile in any three defined patterns were categorized in group unnamed, those with highest score just in one derived patterns named as before. Those who got highest scores in more than on dietary patterns categorized as combined dietary patterns. Totally eight new groups as new dietary patterns were created. ANOVA and ANCOVA were used to compare dependent variables among these new patterns. Also, we tested another factor extracting by calorie adjusted intakes in which each food groups expressed per 1000 Kcal to declare if the same factor loading and factor scores or the same characteristics of dietary patterns would be derived.

## Results

Factor analysis revealed 3 major dietary patterns (Table 1). Food groups with absolute factor loadings >0.20 were considered as having significant contribution to the pattern. These 3 dietary patterns explained 29.02% of the total variance in food intake.

**Table 1:** Factor loading matrix for major dietary patterns identified by factor analysis (n = 734)

**Food items**	**Western like**	**Asian like**	**Traditional like**
Sweet	0.691		
Fast food	0.612		
Drink	0.560		
Red meat	0.489		
Mayonnaise	0.489		
Nuts	0.425	0.394	
Refined grains	0.386		-0.272
Potato	0.375	0.319	-0.307
Visceral meats	0.367		
Vegetables		0.635	0.453
Low fat dairy		0.612	
Fish poultry		0.509	0.233
Eggs		0.422	
High fat dairy	0.247		0.519
Liquid oil	0.269		0.512
Whole grain	-0.219		0.493
Solid oils	0.235		-0.408
Fruits	0.374	0.280	0.403
Pickles		0.291	-0.325
Percentage of variance explained (%)	13.54	8.81	6.66

Omitted from the table were food groups with factor loading < ±0.30 for all dietary patterns.

The characteristics of patients by tertile of dietary pattern score were shown in Table 2. Multivariate linear regression models of dietary pattern 1 were presented in Table 3. Other two dietary patterns did not show any significant relation with studied dependent variables in regression models.

**Table 2:** Characteristics of patients by tertiles of three dietary patterns^*^

	**T1**	**T2**	**T3**	P^**^	P^***^
Age(yrs)	57 ± 6	55 ± 7	52 ± 6	<0.001	<0.001
	55 ± 10	55 ± 10	55 ± 9	0.89	0.77
	52 ± 9	54 ± 11	57 ± 9	0.002	0.002
Duration of diabetes (month)	134 ± 31	128 ± 27	114 ± 20	0.19	0.07
	138 ± 66	124 ± 63	114 ± 86	0.90	0.03
	127 ± 97	119 ± 89	134 ± 98	0.41	0.47
Calorie intake (Kcal/kg)	26 ± 4	29 ± 5	37 ± 7	<0.001	<0.001
	27 ± 11	30 ± 11	36 ± 13	<0.001	<0.001
	29 ± 7	28 ± 8	36 ± 10	<0.001	<0.001
Cholesterol intake (mg/day)	146 ± 63	164 ± 59	229 ± 98	<0.001	<0.001
	145 ± 58	174 ± 60	220 ± 105	<0.001	<0.001
	172 ± 69	174 ± 76	193 ± 100	0.06	<0.001
Fiber intake (g/day)	26 ± 4	28 ± 4	34 ± 5	<0.001	<0.001
	24 ± 8	28 ± 10	37 ± 16	<0.001	<0.001
	26 ± 6	27 ± 6	36 ± 11	<0.001	<0.001
Fasting blood glucose (mg/dl)	163 ± 62	169 ± 75	179 ± 59	0.32	0.13
	182 ± 71	159 ± 65	170 ± 61	0.08	0.26
	174 ± 73	177 ± 68	162 ± 59	0.35	0.35
HbA1c (%)	8.1 ± 1.8	7.8 ± 1.9	8.3 ± 2.1	0.14	0.75
	7.5 ± 1.6	7.5 ± 2.3	7.5 ± 1.9	0.99	0.90
	7.5 ± 2.2	7.6 ± 2.1	7.4 ± 1.5	0.72	0.82
Serum triglyceride (mg/dl)	164 ± 98	160 ± 97	172 ± 130	0.76	0.63
	159 ± 111	161 ± 91	172 ± 123	0.61	0.36
	160 ± 106	173 ± 127	164 ± 96	0.77	0.80
Serum total cholesterol (mg/dl)	161 ± 34	163 ± 43	172 ± 47	0.18	0.07
	165 ± 38	158 ± 37	173.3 ± 48	0.07	0.22
	166 ± 49	167 ± 41	165 ± 36	0.96	0.98
HDL (mg/dl)	49 ± 12	54 ± 68	48 ± 13	0.61	0.92
	48 ± 13	47 ± 11	56 ± 67	0.33	0.22
	44 ± 11	55 ± 70	49 ± 12	0.39	0.73
LDL (mg/dl)	85 ± 23	87 ± 24	94 ± 29	0.08	0.03
	89 ± 23	83 ± 23	94 ± 29	0.03	0.20
	91 ± 30	90 ± 24	87 ± 24	0.70	0.60

*The mean of all variables were presented by Western like, Asian like and Traditional like dietary patterns respectively.

**p-value of analysis of variance.

***p-value of analysis of regression.

**Table 3:** Multivariate linear regression models of western like dietary pattern

	**Model 1**		**Model 2**		**Model 3**	
	**B ± SE**	**P value**	**B ± SE**	**P value**	**B ± SE**	**P value**
FBS	11.03 ± 5.65	0.05	14.73 ± 5.97	0.01	12.49 ± 5.99	0.03
HbA1c	0.12 ± 0.15	0.42	0.16 ± 0.16	0.33	0.12 ± 0.16	0.47
TG	11.24 ± 9.92	0.25	15.81 ± 10.52	0.13	15.76 ± 10.75	0.14
TC	10.11 ± 3.53	0.005	9.52 ± 3.75	0.01	8.71 ± 3.81	0.02
HDL	2.05 ± 3.96	0.60	0.09 ± 4.20	0.98	1.09 ± 4.27	0.79
LDL	5.90 ± 2.21	0.008	5.48 ± 2.36	0.02	5.04 ± 2.40	0.04

Model 1 was adjusted for Age, sex, diabetes duration, type and drug dosage.

Model 2 was adjusted for Age, sex, diabetes duration, type and drug dosage, calorie intake.

Model 3 was adjusted for Age, sex, diabetes duration, type and drug dosage, calorie intake, physical activity.

Table 4 represents the comparison of mean values in each new group. After adjusting for confounders, no significant differences in glycated hemoglobin and lipid profile were seen among 8 new dietary patterns. Only mean value of blood sugar in semi Western-semi Asian dietary pattern was significantly higher than traditional pattern (p = 0.04).

**Table 4:** Comparison of mean values among eight new dietary patterns^*^

	**1(33%)**	**2(12.9%)**	**3(12%)**	**4(12.9%)**	**5(8.2%)**	**6(7.7%)**	**7(8.9%)**	**8(4%)**	p-value^**^
Duration of diabetes (month)	125 ± 82	118 ± 110	126 ± 106	162 ± 95	109 ± 82	130 ± 113	115 ± 87	87 ± 64	0.09
Calorie intake (Kcal/kg)	24.7 ± 7.8	30.4 ± 10.3	30.2 ± 11.1	28.3 ± 8.8	38.6 ± 9.8	41.6 ± 16.5	37.1 ± 12.1	49.1 ± 11.0	<0.0001
Dietary cholesterol (mg/day)	138 ± 46	199 ± 70	194 ± 75	143 ± 43	244 ± 84	216 ± 60	191 ± 80	313 ± 186	<0.0001
Dietary fiber (g/day)	22 ± 7	27 ± 7	29 ± 10	28 ± 8	34 ± 14	33 ± 12	40 ± 18	54 ± 14	<0.0001
FBS(mg/dl)	174 ± 75	172 ± 61	161 ± 64	154 ± 62	197 ± 62	177 ± 62	162 ± 59	169 ± 47	0.47
HbA1c(%)	7.6 ± 2.2	7.1 ± 1.7	7.3 ± 2.3	7.3 ± 1.3	8.1 ± 1.8	7.7 ± 1.7	7.4 ± 1.5	7.1 ± 1.5	0.63
TG(mg/dl)	163 ± 89	169 ± 149	155 ± 104	151 ± 77	192 ± 158	144 ± 63	179 ± 131	184 ± 87	0.85
Serum total cholesterol (mg/dl)	163 ± 40	163 ± 41	164 ± 45	158 ± 34	188 ± 62	166 ± 31	165 ± 36	180 ± 46	0.27
LDL-C(mg/dl)	87 ± 25	88 ± 24	88 ± 25	81 ± 19	105 ± 35	89 ± 24	88 ± 23	98 ± 33	0.09
HDL-C(mg/dl)	47 ± 11	46 ± 13	70 ± 21	49 ± 12	51 ± 13	52 ± 12	47 ± 11	49 ± 13	0.39

*1 unnamed, 2 Western like dietary pattern, 3 Asian like dietary pattern, 4 Traditional like dietary pattern, 5 Western like-Asian like dietary pattern, 6 western like-Traditional like dietary pattern, 7 Asian like-Traditional like dietary pattern and 8 western like-Asian like-Traditional like dietary pattern.

**p-value of analysis of variance.

Calorie adjusted factor extracting showed three major patterns with different characteristics and factors loading that explained 25.17% of the total variance in food intake (Table 5). Factor 1 and 2 showed some characteristics of Western and Asian like dietary patterns respectively. But, there were many differences between factor 3 (characterized by high loading of refined grains-solid oils) and derived Traditional like dietary pattern.

**Table 5:** Factor loading matrix for major dietary patterns identified by factor analysis on calorie adjusted food groups (n = 734)

**Food items**	**Western like**	**Asian like**	**Refined grains-solid oils**
Drink	0.623		
Fast food	0.568		
Sweet	0.518	-0.211	
Visceral meat	0.503		
Mayonnaise	0.502		
Nuts	0.340		-0.228
Vegetables		.0700	-0.253
Fish poultry		0.565	
Eggs		0.565	
Low fat dairy	-0.208	0.560	
Refined grains			0.620
Liquid oils			-0.558
Solid oils			0.537
potato	0.334		0.396
Whole grains	-0.327		-0.385
Fruits			-0.330
Pickles			0.312
Percentage of variance explained (%)	10.28	7.56	7.23

Omitted from the table were food groups with factor loading < ±0.30 for all dietary patterns.

## Discussion

### Some patients may show combined dietary patterns

The new statistical approach to approximate dietary patterns based on classification of individuals according to the highest scores in each pattern (unlike the conventional analysis), showed some individuals may comply combined dietary patterns which could affect the results of associations in comparison to considering them only in one dietary pattern. As we showed, by conventional analysis, the high adherence to Western like dietary pattern may be associated with glycemia and serum lipid profiles; while comparison the highest tertiles of eight new dietary patterns showed no differences except increased serum glucose in combined Western like-Asian like but not in Western like separately. So, the results of a new dietary pattern analysis method could have controversies with commonly used method. As in this study, the conventional dietary pattern analysis showed a positive association between Western like dietary pattern and blood lipids and glucose, while this association was not seen in new classification. Since, dietary pattern interactions were not considered in published papers [5-9]. For example, when the health effects of Mediterranean dietary pattern are attributed to its antioxidant properties [10], the question arise that if a person have higher score in both Mediterranean and also another dietary pattern with high pro-oxidant properties, could we expect the same Mediterranean’s dietary pattern effects as well? Or, even if this person has higher score in both Mediterranean dietary pattern and another with high antioxidant properties besides its other beneficial effects, could we expect synergistic effects, too?

### Primary calorie adjusted factor analysis shows some different dietary patterns

Another point of controversy may arise, as shown in our study, is when factors score of each food group is determined for participant according to the total amount of that group intake not based on calorie intake. In the other hand, neglecting of calorie intake in determining the loading factor and factor scores of dietary patterns could create a substantial inaccuracy in classification of people in derived dietary patterns. Adjusting for calorie intake often performed in afterward analysis such as regression, ANCOVA and etc. So, when we have a group of subjects with different calorie requirements, certainly different serving sizes would expected. While in dietary pattern analysis a person who consumes more scores more regardless of calorie intake and consequently food requirements.

### Interpretation of dietary pattern analysis based on food items may challenge the basic concept of factor analysis

Another question is that, to what extent was dietary pattern analysis successful in innovating new ideas? What have been the new outcomes? As seen in published papers, we almost always back to the basic knowledge in explanation of the effects or associations of dietary patterns. For example, comparison the effects of different diets in a systematic review have revealed that dietary patterns containing fiber-rich foods have a protective role in managing diabetes mellitus [5]. In other words, what extent of inconsistency exists between the results of related dietary pattern and nutrient based studies? When we show dietary patterns characterized by high consumption of fruit and vegetables, whole grains, fish, and poultry, and by decreased consumption of red meat, processed foods, sugar-sweetened beverages, and starchy foods may retard the progression of type 2 diabetes [6-8], or patterns including whole grains, legumes, vegetables, and fruits could improve lipid profiles [9], what would be the new outcome in regard to what we knew. While, our expectation of new analysis method which named dietary pattern analysis was to consider whole dietary intake in a complex form not just considering food items or nutrients separately. Does getting relied purely on each food and nutrient properties in interpretation the effects of derived dietary patterns could not challenge the basic concept of factor analysis?

This is the first study represents and explains the single or combined dietary patterns and calorie adjusted factor analysis as better method to derive and interpret associated results and could bring an evolution dietary pattern analysis. This can be taken to account as the strengths of this study.

This study also has several limitations such as subjective decision making for determination of number of patterns, the method of rotation and labeling the patterns can be considered as a limitation of factor analysis.

## Conclusion

It seems that conventional dietary pattern analysis method has some limitations which should be seriously considered. In order to achieve more accurate results, it is recommended derive combined dietary patterns according to the persons’ adherence to each pattern.

## Abbreviations

ANCOVA: Analysis of covariance; ANOVA: Analysis of variance; FFQ: Food frequency questionnaire; HbA1c: Hemoglobin A1C; HDL: High density lipoprotein; KMO: Kaiser-Meyer-Olkin; LDL: Low density lipoprotein; MET: Metabolic equivalent; PCA: Principal component analysis; SPSS: Statistical package for the social sciences; TG: Triglyceride; TC: Total cholesterol.

## Competing interests

There are not any financial or non-financial competing interests to declare in relation to this manuscript.

## Authors’ contributions

ZS conceived of the study, carried out its designing, coordinated the implementation, drafted the manuscript, and performed the statistical analysis. MA participated in acquisition of data and revised the manuscript. NP participated in acquisition of data and revised the manuscript. BL participated in the design of the study and revised the manuscript. MQ participated in analysis and interpretation of data and revised the manuscript. MK participated in the design of the study and revised the manuscript. All authors read and approved the final manuscript.
